# Inflammatory Profile and Risk of Hypertension in Infants Following Coarctation of the Aorta Repair: The Role of IL-6/TNF-α Ratio

**DOI:** 10.3390/life15091481

**Published:** 2025-09-21

**Authors:** Irina-Maria Margarint, Vlad Anton Iliescu, Tammam Youssef, Iulian Rotaru, Alexandru Popescu, Olguta Untaru, Radu Vladareanu

**Affiliations:** 1 Faculty of Medicine, Carol Davila University of Medicine and Pharmacy, 050474 Bucharest, Romania; irina-maria.margarint@drd.umfcd.ro (I.-M.M.); tammyoussef25@gmail.com (T.Y.); vladareanu@gmail.com (R.V.); 2 Department of Cardiac Surgery, Emergency Clinical Hospital for Children “Maria Skłodowska Curie”, 077120 Bucharest, Romania; rotaru.iulian7@yahoo.com (I.R.); alexandru.a.g.popescu@gmail.com (A.P.); untaru_olguta@yahoo.com (O.U.)

**Keywords:** aortic coarctation, inflammation biomarkers, postoperative hypertension, IL-6/TNF-α ratio

## Abstract

Background: Despite anatomically successful surgical correction, postoperative hypertension remains a significant concern in patients with coarctation of the aorta, even when repair is performed during infancy. Inflammation and neurohormonal activation have been proposed as contributing mechanisms. Objective: To investigate the association between preoperative inflammatory biomarkers—specifically the interleukin-6 (IL-6) to tumor necrosis factor-alpha (TNF-α) ratio—and the development of hypertension in patients with successful isolated coarctation of the aorta repair under one year of age. Methods: This observational study included 42 infants with isolated CoA. Clinical, echocardiographic, and biochemical parameters were analyzed. Preoperative plasma levels of IL-6, TNF-α, von Willebrand factor (vWF), and renin were measured. Patients were classified based on hypertensive status at 2-year follow-up. Univariate and multivariate logistic regression analyses were performed to identify predictors of postoperative hypertension. Results: Hypertension was diagnosed in 16 out of 41 patients (39%) at follow-up. A preoperative IL-6/TNF-α ratio > 2 was an independent predictor in multivariate analysis for postoperative HT (OR = 6.1, 95% CI = 6.23–9.31, *p* = 0.02). Conclusions: In this small single-center cohort, an elevated IL-6/TNF-α ratio was associated with postoperative hypertension after coarctation repair. These exploratory findings should be considered hypothesis-generating and warrant confirmation in larger, multicenter studies.

## 1. Introduction

Coarctation of the aorta is one of the most frequent congenital malformations, with an incidence of 5% to 7% [1]. Transcatheter techniques and surgical management strategies are well established and offer good results in the immediate postoperative period [2]. Long-term mortality and morbidity are not, however, well understood, since aortic coarctation is no longer seen as a mechanical obstruction of the aorta but rather a systemic condition with mechanisms like endothelial dysfunction, aortic wall changes, and an inflammatory process that contributes to hypertension development later in life [3]. Hypertension (HT) after aortic coarctation (CoA) repair has an incidence of 20% to 40% and contributes to shorter life expectancy due to cardiovascular and cerebrovascular events [3]. One of the mechanisms involved in HT after CoA repair is elevated proinflammatory cytokines, a state that influences vascular reactivity and promotes endothelial dysfunction and the atheromatous process [4]. Both interleukin-6 (IL-6) and tumor necrosis factor alpha (TNF) are inflammatory cytokines reported to have high levels in pediatric patients after CoA repair [5,6]. Moreover, IL-6 is reported to increase more than TNF in inflammatory systemic states, and a higher IL-6/TNF ratio could better reflect this process [7,8]. The study aims to evaluate if IL-6/TNF ratio is associated with HT occurrence after successful CoA repair.

## 2. Materials and Methods

Patients with severe aortic coarctation diagnosed by transthoracic echocardiography were referred to our institute (Emergency Clinical Hospital for Children “Maria Skłodowska Curie”, Bucharest, Romania) between January 2022 and December 2022. Patients data were collected from the electronic system of our center.

The inclusion criteria were: severe aortic coarctation based on transthoracic echocardiography examination. Exclusion criteria were the following: (1) age greater than one year; (2) other significant congenital heart defects with indications for surgery; (3) surgical contraindications; (4) underlying medical conditions with adverse short-term outcomes; and (5) underlying disorders leading to arterial hypertension (e.g., renal disease).

Transthoracic echocardiography (TTE) was used for the evaluation of these patients (Philips EPIQ CVX Cardiology Ultrasound System). Assessment of the aortic arch was performed from the suprasternal notch, focusing on anatomical configuration, vessel diameters, and flow gradients derived from Doppler imaging. Color Doppler was utilized to identify the precise location of the coarctation and to detect flow disturbances caused by the stenotic lesion. Both pulsed-wave and continuous-wave Doppler modalities were applied to characterize the flow profile within the descending thoracic aorta, determine peak systolic velocities, and estimate the maximum instantaneous pressure gradient across the narrowed segment. Aortic coarctation was considered hemodynamically significant if the peak gradient measured at the isthmus exceeded 20 mmHg, in conjunction with diastolic “runoff” and a luminal narrowing greater than 50% compared to sex- and BMI-adjusted normative values, or a Z-score less than −3 at the isthmic level.

Pre-surgical evaluation involved thoracic cardiac computed tomography (CT) imaging. This allowed for comprehensive visualization of the aorta and its branches, assessment of collateral circulation, and analysis of anatomical relationships with adjacent extracardiac structures.

Prior to surgery, fasting blood samples were obtained after a minimum of 10 h of fasting, to determine circulating levels of renin, aldosterone, interleukin-6 (IL-6), tumor necrosis factor-alpha (TNF-α), and von Willebrand factor (vWF). All measurements were conducted in the morning, with patients in the supine position. Reference ranges were established according to the standards of our institution’s clinical laboratory: renin 3–28 pg/mL (supine), aldosterone 2.21–35.3 ng/dL (supine), IL-6 < 7 pg/mL, vWF 50–150 UI/mL, and TNF-α < 8.1 pg/mL. For the IL-6/TNF-α ratio, we applied the criteria proposed by Angelone et al., to define an increased IL-6/TNF ratio. In his study, a ratio of IL 6/TNF greater than the adult median plus 2 standard deviations was interpreted as significant for systemic inflammation, with corresponds to a ratio greater than 2 [9].

An extended end-to-end anastomosis was achieved via a left thoracotomy, with all cases, performed by the same surgeon.

Follow-up was performed over a period of 2 years. The protocol of blood pressure measurement is the same used in a study performed by our group, which reported that increased plasma levels of renin are independently associated with the occurrence of postoperative HT in patients with successful CoA repair [3]. Blood pressure was measured in the right upper limb during scheduled follow-up visits at 3, 6, 12, and 24 months postoperatively, using an automated oscillometric monitor (Philips Intellivue MP30, Philips Healthcare, Best, Netherlands) with pediatric cuffs appropriately adapted to arm length and circumference. All measurements were performed in a calm setting, with the patient in a resting supine position for at least five minutes before recording. To ensure accuracy and reproducibility, three consecutive measurements were obtained at 15 min intervals during each visit.

For clinical assessment, the average of the three blood pressure measurements was considered. A single, standardized monitoring device was utilized consistently at all time points across all participants. The diagnosis of hypertension (HT) adhered to the 2017 Clinical Practice Guidelines issued by the American Academy of Pediatrics (AAP), which define HT as systolic and/or diastolic blood pressure values equal to or exceeding the 95th percentile for age, sex, and height on a minimum of two separate follow-up evaluations, or normotensive readings in the context of ongoing antihypertensive therapy. Percentile cutoffs were determined using the reference charts provided in the AAP guidelines, adjusted for age, sex, and height. Patients were classified as hypertensive if all three recorded values during follow-up surpassed these thresholds.

### Statistical Analysis

Statistical analysis was performed using Wizard 2 Statistical Software for Mac OS (Wizard–Statistics & Analysis, Raipur, Chhattisgarh, India). Categorical variables are expressed as absolute frequencies and percentages, while continuous variables are reported as means ± standard deviations. The primary endpoint was the incidence of postoperative hypertension (HT).

To explore the association between the IL-6/TNF-α ratio and the development of HT following coarctation of the aorta (CoA) repair, a multivariable logistic regression model was applied. The model incorporated variables that demonstrated a *p*-value < 0.1 in univariate analysis. A backward stepwise selection strategy was then employed for predictive modeling.

The following variables were assessed in the univariate analysis: patient age, weight, height, preoperative aortic isthmus diameter, Z-score at the isthmus, prematurity status, presence of moderate or severe left ventricular dysfunction, bicuspid aortic valve, aortic arch morphology (crenel, gothic, or Roman), proximal and distal aortic arch diameters, preoperative and postoperative Doppler-derived peak gradients and velocities at the isthmus, need for inotropic or vasoactive support, elevated preoperative serum levels of renin, aldosterone, IL-6, vWF, and TNF-α (above institutional reference thresholds), and aortic cross-clamp time.

Results of the logistic regression analysis are presented as odds ratios (OR) with corresponding 95% confidence intervals and associated *p*-values.

## 3. Results

### 3.1. Patient Characteristics

**Figure 1:**
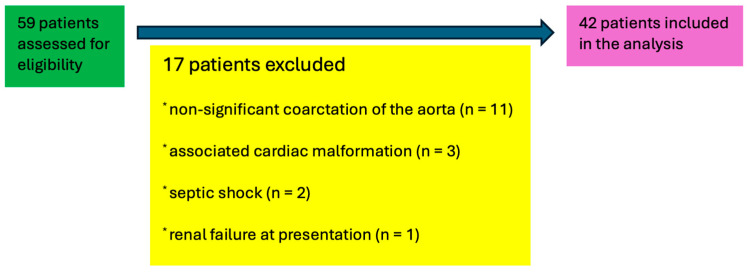
Patients included in the study after applying the exclusion criteria.

In total, 59 patients were transferred to our center. After the exclusion criteria were applied, 42 patients were included in the study. Aortic coarctation without hemodynamically significance as defined earlier (cases with a Doppler-derived peak gradient < 20 mmHg at the isthmus and/or less than 50% narrowing relative to normal reference values (Z-score > −3), without clinical or imaging evidence warranting surgical repair) was present in 11 patients; 3 patients had associated congenital heart defects: one ventricular septal defect, one double outlet right ventricle, and one complete atrioventricular canal. 2 patients were with severe septic shock, and one patient was with associated renal malformation and renal disease. (Figure 1).

Symptoms at presentation were the following: pressure gradient over 20 mmHg between upper and lower limbs in 82.92%, difficulty feeding in 51%, oliguria in 60.97%, tachypnea in 85.36% and in 73.17% of cases, pulse oximetry values were decreased in the lower limbs compared to the upper limbs.

Table 1 highlights the characteristics of the patients included in the study. Only patients under one year were included in the study, with a mean age of 5.5 ± 3.67 m, and 6 patients were born prematurely. The mean weight was 5024.1 ± 2214.73 g, the mean height was 63.7 ± 9.72 cm, 87.8% of patients were of male sex. Severe left ventricular dysfunction was present in 9.8% of cases, 7.3% of patients required preoperative inotropic support, while in 14.6% of cases, both inotropes and vasopressor agents were used. Table 2 presents the comparison of patients with and without postoperative hypertension. There were no significant differences between pre-/intra-/postoperative characteristics of the two groups.

### 3.2. Coarctation of the Aorta—Characteristics

The mean diameter of the coarctation zone was 0.95 ± 0.17 mm with a mean Z score of−3.85 ± 1.16. The Gothic arch was the most frequent particular arch anatomy encountered in 29.3% of cases, there were 5 cases of bovine arch (12.2%), and 3 patients with a crenel type of aortic arch. The mean diameter of the ascending aorta was 8.41 ± 1.76 mm, proximal mean diameter of the aortic arch was 6.98 ± 2 mm, the mean diameter of the distal arch was 4.8 ± 1.34 mm, and a bicuspid valve was present in 14.6% of patients. The mean peak systolic gradient and peak velocity at the coarctation zone were 38.31 ± 23.2 mmHg and 2.97 ± 0.87 m/s, respectively.

The mean duration of surgery was 96.2 ± 20.71 min, and aortic clamp time was 28.95 ± 7.97 min. Surgery under one month of age was performed in 9.75% of cases. One patient required the use of extracorporeal circulation due to preoperative severe cardiogenic shock. Postoperative peak systolic gradient and velocity at the isthmus were 12 ± 6.27 mmHg and 1.7 ± 0.4 m/s, respectively. Postoperative complications were the following: one patient developed a chylothorax, one patient developed a superficial wound infection, and three patients evolved with cardiogenic shock. There were no postoperative deaths.

Incidence of postoperative HT was 39% (16 patients). Patients with HT have a significant incidence of prematurity (*p* = 0.03), longer aortic clamp time (*p* = 0.039), higher levels of IL-6 (*p* < 0.001), and a higher incidence of IL-6/TNF-alpha ratio over 2 (*p* < 0.001).

### 3.3. Inflammatory Biomarkers

**Figure 2:**
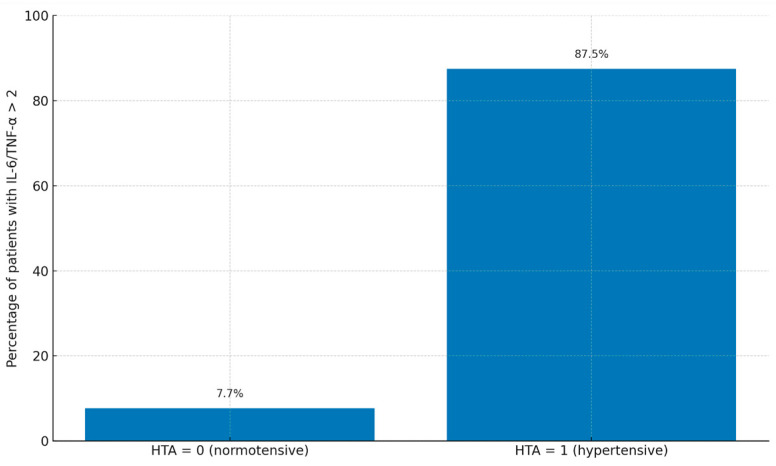
Bar chart showing the proportion of patients with IL-6/TNF-α ratio > 2 stratified by postoperative hypertension status (HTA 0 = normotensive, HTA 1 = hypertensive). Percentages are displayed above each bar.

The mean IL-6 and TNF-alpha plasma concentrations were 54.52 ± 64.77 pg/mL and 30.5 ± 32.08 pg/mL, respectively. A IL-6/TNF-alpha ratio over 2 was seen in 39% of cases. Increased levels of IL-6 and TNF-alpha were observed in 85.4% and 95.1%, respectively. Higher levels of renin plasma concentrations were seen in 78% of patients with a mean renin plasma concentration of 56.32 ± 61.24 pg/mL. Also, elevated concentrations of vWF were observed in 12.2% of cases with a mean plasma concentration of 77.9 ± 63.13. There were significant differences between the mean values of plasma concentrations of IL6 (4.5 ± 1.8 vs. 6.9 ± 2.3, *p* = 0.02), renin (22 ± 7 vs. 35 ± 10, *p* = 0.01), TNF (4.8 ± 1.7 vs. 7.2 ± 2.1, *p* = 0.01) and vWF (140 ± 30 vs. 170 ± 40, *p* = 0.03) between patients with and without postoperative hypertension. Figure 2 illustrates the proportion of patients with an IL-6/TNF-α ratio > 2 stratified by postoperative hypertension status. A significantly higher percentage of patients in the hypertensive group exhibited elevated IL-6/TNF-α ratios compared to the normotensive group (*p* = 0.03).

### 3.4. Logistic Regression

Univariate analysis (Table 3) was first performed for all clinical and biomarker variables stated in the Methods section. Variables with *p* < 0.1 (Renin plasma concentration, Gothic arch, Aortic clamp time, Age), were included in a multivariable logistic regression model, which was subsequently reduced using backward stepwise selection. Multicollinearity was checked by variance inflation factors (all < 2), and regression diagnostics were performed to verify model fit. Given the exploratory design, no adjustments for multiple comparisons were applied. Table 2 presents the outcomes of the univariate analysis for variables with a *p*-value below 0.1. Among these, elevated plasma renin concentration (OR = 3.41, 95% CI: 2.6–4.73, *p* = 0.05) and prolonged aortic clamp time (OR = 3.76, 95% CI: 2.9–6.1, *p* = 0.04) were retained in the final multivariable model following backward stepwise selection. An IL-6/TNF-α ratio > 2 demonstrated a strong association with hypertension in univariate analysis (OR = 8.23, 95% CI: 5.57–12.4, *p* < 0.001) and remained an independent predictor of postoperative hypertension after model adjustment (OR = 6.1, 95% CI: 4.2–9.31, *p* = 0.02). The multivariable model explained 42.3% of the variance in postoperative hypertension risk (Nagelkerke R^2^ = 0.423) and showed good predictive performance, correctly classifying 85.3% of hypertensive (HT+) and 76.4% of normotensive (HT–) patients, with an overall accuracy of 81.2%. When IL-6 and TNF-α were introduced as separate continuous variables in the multivariable model, predictive accuracy decreased (overall classification accuracy = 73.5%). When dichotomized by individual medians or quartiles, performance remained inferior to using the IL-6/TNF-α > 2 threshold. This supports the utility of the combined cytokine ratio as a better predictor of hypertensive phenotype in our cohort.

## 4. Discussion

Coarctation of the aorta (CoA) is a congenital malformation characterized by a narrowing of the thoracic aorta, most commonly situated just distal to the origin of the left subclavian artery. It represents approximately 5–8% of all congenital heart anomalies and has an estimated incidence of 4 per 10,000 live births [9].

CoA is frequently associated with other congenital anomalies, such as bicuspid aortic valve, ventricular septal defect, or Turner syndrome. Early diagnosis and treatment—whether surgical or interventional—are critical, as untreated cases carry a high risk of left ventricular failure, aortic rupture, and death in infancy [10].

CoA is no longer seen just as a simple narrowing of the aorta but rather as a systemic disease [3]. Despite anatomically successful repair, systemic arterial hypertension remains a common long-term complication, reported in 20% to 40% of pediatric patients after CoA repair [11,12]. This elevated blood pressure can persist into adolescence and adulthood, increasing the risk of premature cardiovascular morbidity. The underlying mechanisms are multifactorial and remain incompletely understood. Vascular dysfunction, structural alterations of the aortic wall leading to increased stiffness, and chronic inflammatory processes may act independently or synergistically to promote the development of hypertension after coarctation of the aorta repair [3].

Several studies have demonstrated the presence of endothelial dysfunction post-repair, as evidenced by impaired flow-mediated vasodilation and increased arterial stiffness [13,14,15,16]. Divitiis et al. and Trojnarska et al. reported reduced endothelium arterial relaxation and nitroglycerine-mediated vasodilatation impairment in the brachial artery, reflecting an impaired capacity of smooth vascular muscle to relax [13,17]. Furthermore, structural modifications of the aortic wall, including reduced elasticity, increased collagen deposition, and abnormal media thickness, have been described. Ou P et al., Shang Q et al., and Rog B et al. all reported decreased central aortic distensibility and increased stiffness [18,19,20], while Cetiner N et al. reported these findings in the abdominal aorta and at the peripheral level at the brachial artery [21]. Also, particular anatomy of the aortic arch seems to be linked to increased central aortic stiffness, especially the gothic type [22].

Chronic low-grade inflammation has been increasingly recognized as a key player in perpetuating vascular dysregulation following CoA repair, particularly through its effects on endothelial activation, arterial tone regulation, and autonomic imbalance [23,24]. Among the proinflammatory cytokines involved in vascular inflammation, IL-6 and tumor necrosis factor TNF-α play central roles. IL-6 is produced by activated monocytes, endothelial cells, and fibroblasts and contributes to sympathetic nervous system stimulation, sodium retention, and angiotensin II receptor upregulation [15,25]. TNF-α is a potent mediator of endothelial activation, promoting leukocyte adhesion, apoptosis, and oxidative stress [26]. In conditions of systemic inflammation, the balance between these two cytokines shifts. The IL-6/TNF-α ratio has been used in pediatric and neonatal research as a functional marker of inflammatory polarization, with higher values indicating a dominance of IL-6-mediated responses. For example, Angelone et al. showed that neonates have a physiologically elevated IL-6/TNF-α ratio compared to adults, and ratios > 2 may suggest biologically significant proinflammatory state [27]. In juvenile idiopathic arthritis, Cohen et al. reported IL-6/TNF-α ratios between 2 and 10 [28], and Ríos et al. found ratios > 5 to 15 to be associated with severe forms of MIS-C in pediatric COVID-19 patients [29,30].

In the following section, we distinguish between mechanisms supported by established evidence and those proposed here as exploratory hypotheses

In our cohort, IL-6 was elevated in 85.4% and TNF-α in 95.1% of patients under one year of age who underwent CoA repair. Notably, 39% of patients had an IL-6/TNF-α ratio > 2, suggesting an imbalance favoring IL-6-dominated inflammatory responses. IL-6, more than TNF-α, may drive endothelial dysfunction and altered vascular reactivity associated with postoperative hypertension. This is of particular interest, as recent studies suggest that a higher IL-6/TNF ratio is associated with impaired vasodilation, increased arterial stiffness, and elevated sympathetic tone—mechanisms that can contribute to persistent or recurrent hypertension after aortic arch intervention [31,32,33,34].

Although von Willebrand factor (vWF), a marker of endothelial activation, showed significant differences between hypertensive and normotensive patients, it was not associated with postoperative hypertension, while the IL-6/TNF-α ratio remained independently associated with hypertension in multivariate analysis. This may indicate that the inflammatory dysregulation is not necessarily reflected in vWF dynamics in this population, or that IL-6/TNF is a more sensitive marker in the early postoperative phase. However, renin levels differ significantly between the two groups of patients and were associated with postoperative hypertension, results reported by our group in a previous study [3]. Our present findings support the concept that early postoperative hypertension in infants undergoing coarctation repair is a multifactorial phenomenon, shaped by the interplay between neurohormonal activation and systemic inflammation. While our previous work demonstrated a significant association between elevated plasma renin activity and late-onset hypertension in similar patients [3], the current study emphasizes the independent predictive role of inflammatory imbalance—specifically, an IL-6/TNF-α ratio > 2—as a stronger correlate of early hypertensive phenotype. These differences suggest that RAAS activation, endothelial dysfunction, and cytokine-driven vascular reactivity may act through overlapping yet temporally distinct mechanisms. The persistent influence of inflammatory mediators, even in the presence of elevated renin levels, highlights the need to consider immune–neuroendocrine interactions when evaluating postoperative blood pressure trajectories in this vulnerable population.

Our findings on the IL-6/TNF-α imbalance can be interpreted in the broader context of vascular remodeling and extracellular matrix turnover. Recent evidence by Costa et al. [35] highlights the dual role of matrix metalloproteinases (MMPs) as both molecular effectors of vascular pathology and sociomarkers reflecting broader psychosocial determinants of health. This framework suggests that postoperative hypertension after coarctation repair may arise not only from inflammatory imbalance but also from concomitant extracellular matrix remodeling. Integrating cytokine markers with matrix-remodeling enzymes such as MMPs may therefore provide a more comprehensive and predictive biomarker strategy for vascular dysfunction and postoperative outcomes. This multimarker approach could help identify high-risk patients early and guide more tailored interventions.

These findings suggest that the IL-6/TNF-α ratio may serve not only as a marker of inflammation but also as a predictive tool for identifying patients at risk of postoperative hypertension after CoA repair. This could allow for more personalized follow-up strategies, early antihypertensive therapy, and possibly the use of targeted anti-inflammatory interventions in high-risk patients. In our study, the cutoff value of IL-6/TNF-α ratio > 2 was adopted from previous neonatal research, as no validated threshold currently exists for pediatric patients undergoing coarctation repair. We acknowledge this as a limitation, and therefore interpret our findings as exploratory. Nevertheless, the consistent association of this marker with postoperative hypertension in our cohort suggests biological plausibility and highlights the need for larger prospective studies to establish population-specific reference values. The interplay between inflammation and vascular tone regulation may represent an additional mechanism underlying residual hypertension after CoA repair. Endothelin-1, nitric oxide, and serotonin are known modulators of vascular reactivity, and their imbalance has been implicated in the development and persistence of high blood pressure, even in the absence of structural heart disease [36]. In this context, an elevated IL-6/TNF-α ratio may act synergistically with dysregulated vasoactive pathways, amplifying vascular dysfunction and sustaining postoperative hypertension. Future studies simultaneously assessing inflammatory and endothelial mediators may better identify subgroups at highest risk and provide a more comprehensive characterization of disease mechanisms.

Limitations of this study include the relatively small sample size and the limited duration of follow-up. Although patients were observed over a period of 2 years, this may not fully capture the long-term cardiovascular trajectory of these patients. Another limitation is the lack of serial postoperative cytokine measurements, which would provide valuable insight into the temporal association between inflammation and the development of postoperative hypertension. Also, because cytokines were assessed at a single preoperative time point, our data cannot fully capture their dynamic fluctuations; therefore, conclusions regarding a chronic inflammatory state are limited, and future studies with repeated measurements are warranted. The use of a stringent definition of postoperative hypertension (elevated values at all three follow-up visits) can be another limitation, but sensitivity analysis applying the guideline minimum of ≥2 visits yielded similar associations, supporting the robustness of our findings. The use of a stricter definition of postoperative hypertension may have excluded patients with intermittent or borderline hypertension. Ambulatory blood pressure monitoring, which is considered more accurate in capturing variability and masked hypertension in children, was not available in our study, representing a limitation. Given the limited sample size, our regression analysis may be subject to unstable estimates and an increased risk of type I error. Although the limited sample size raises the possibility of overfitting in our multivariable model, the analysis should be viewed as exploratory, providing a rationale for future studies that may apply penalized regression or simplified models to confirm these associations. Therefore, the results should be interpreted with caution and regarded as hypothesis-generating.

Further validation in multicenter cohorts with extended monitoring is necessary. Additionally, cytokine measurements were taken at a single time point, which may not reflect dynamic changes in inflammatory status over time. From a clinical standpoint, the IL-6/TNF-α ratio is readily obtainable with routine immunoassays and may serve as an adjunctive preoperative risk marker. In settings where a higher ratio is identified, clinicians could intensify peri- and post-operative BP monitoring (including consideration of ABPM), adopt a lower threshold for initiating antihypertensive therapy in the presence of repeated elevated readings, optimize analgesia and limit catecholamine exposure, and schedule closer follow-up. These applications are hypothesis-generating; standardization of assays, age-specific reference ranges, and external validation in larger multicenter cohorts are prerequisites before routine implementation.

## 5. Conclusions

We analyzed a cohort of 41 patients who underwent successful surgical repair of isolated coarctation of the aorta (CoA) within the first year of life. At two-year follow-up, we observed a high incidence of postoperative HT. A preoperative IL-6/TNF-α ratio greater than 2 was independently associated with the development of HT. These findings support the hypothesis that chronic low-grade inflammation may contribute to the pathogenesis of residual hypertension in this population and reinforce the concept that CoA represents not only a mechanical obstruction but rather a systemic vascular disease with inflammatory and endothelial components. Two key clinical implications emerge from our results: (1) the need for long-term blood pressure surveillance in pediatric patients after anatomically successful CoA repair, particularly in those with evidence of systemic inflammation; (2) the IL-6/TNF-α ratio may serve as a valuable prognostic biomarker, identifying a subgroup of patients who could benefit from intensified blood pressure monitoring and early initiation of antihypertensive therapy; and (3) comprehensive, sex-sensitive strategies could include tailored screening for hypertension and vascular biomarkers, integration with metabolic and diabetes management programs, and routine referral to psychosocial or addiction services to address modifiable risk factors. Given the limited sample size and retrospective design, our results should be viewed as preliminary and interpreted with caution, underscoring the need for external validation before clinical application. Further prospective studies are warranted to validate these findings and to improve our understanding of the mechanisms underlying postoperative hypertension following early CoA repair.

## Figures and Tables

**Table 1:** Characteristics of the patients.

Parameter (Unit)	N = 42 (100%)
**Preoperative**	
Age (mean, SD months)	5.5 ± 3.67
Weight (mean, SD grams)	5024.1 ± 2214.73
Height (mean, SD cm)	63.7 ± 9.72
Male sex (n, %)	36 (87.8)
Premature (n, %)	6 (14.6)
Moderate left ventricular disfunction (n, %)	5 (12.2)
Severe left ventricular disfunction (n, %)	4 (9.8)
Diameter of the aorta at the isthmus (mean, SD mm)	0.95 ± 0.17
Z score (mean, SD)	−3.85 ± 1.16
Bicuspid aortic valve (n, %)	6 (14.6)
Gothic aortic arch (n, %)	12 (29.3)
Crenel gothic arch (n, %)	3 (7.3)
Bovine aortic arch (n, %)	5 (12.2)
Ascending aortic diameter (mean, SD mm)	8.41 ± 1.76
Proximal aortic arch diameter (mean, SD mm)	6.98 ± 2
Distal aortic arch diameter (mean, SD mm)	4.8 ± 1.34
Peak systolic gradient at the aortic isthmus (mean, SD mmHg)	38.31 ± 23.2
Peak velocity at the isthmus (mean, SD m/s)	2.97 ± 0.87
Inotropic support (n, %)	3 (7.3)
Inotropic and vasopressor support (n %)	6 (14.6)
**Biomarkers (plasma concentration)**	
Renin (mean, SD pg/mL)	56,32 ± 61.24
Renin over 28 pg/mL (n, %)	32 (78)
vWF (mean, SD UI/mL)	77.9 ± 63.13
vWF over 150 UI/mL (n, %)	5 (12.2)
IL-6 (mean, SD, pg/mL)	54.52 ± 64.77
IL-6 over 7 pg/mL (n, %)	35 (85.4)
TNF-alpha (mean, SD, pg/mL)	30.5 ± 32.08
TNF-alpha over 8.1 pg/mL (n, %)	39 (95.1)
IL-6/TNF-alpha over 2	16 (39)
**Intraoperative**	
Duration of surgery (mean, SD min)	96.2 ± 20,71
Aortic clamp time (mean, SD min)	28.95 ± 7.97
Surgery under one month (n, %)	4 (9.75)
Extracorporeal circulation (n, %)	1 (2.43)
**Postoperative**	
Peak systolic gradient at the aortic isthmus (mean, SD m/s)	12 ± 6.27
Peak velocity (mean, SD m/s)	1.7 ± 0.4
Inotropic and/or vasopressor support (n, %)	4 (9.8)
Complications	5 (12.2)
Chylothorax (n, %)	1 (2.43)
Superficial wound infection (n,%)	1 (2.43)
Cardiogenic shock (n, %)	3 (7.3)
Hypertension (n, %)	16 (39)

**Table 2:** Comparison of patients with and without postoperative hypertension.

Parameter (Unit)	HTA (0) N = 26	HTA (1) N = 16	*p*
**Preoperative**			
Age (mean, SD months)	40.4 ± 41.2	37.7 ± 54.8	0.86
Weight (mean, SD grams)	5400 ± 700	5100 ± 800	0.22
Height (mean, SD cm)	61.2 ± 3.5	59.8 ± 4.0	0.25
Male sex (n, %)	10 (38.46)	16 (100)	0.37
Premature (n, %)	3 (11.5)	3 (18.75)	0.83
Moderate left ventricular disfunction (n, %)	4 (24%)	4 (17%)	0.88
Severe left ventricular disfunction (n, %)	0 (0%)	5 (21%)	0.13
Diameter of the aorta at the isthmus (mean, SD mm)	2.8 ± 0.9	2.7 ± 1.0	0.77
Z score (mean, SD)	−1.3 ± 0.5	−1.6 ± 0.7	0.19
Bicuspid aortic valve (n, %)	6 (35%)	10 (42%)	0.63
Gothic aortic arch (n, %)	5 (29%)	2 (8%)	0.18
Crenel aortic arch (n, %)	3 (18%)	5 (21%)	0.72
Bovine aortic arch (n, %)	2 (12%)	6 (25%)	0.31
Ascending aortic diameter (mean, SD mm)	8.7 ± 2.3	7.6 ± 1.3	0.08
Proximal aortic arch diameter (mean, SD mm)	5.7 ± 1.2	6.0 ± 1.1	0.47
Distal aortic arch diameter (mean, SD mm)	4.7 ± 1.2	5.0 ± 1.1	0.41
Peak systolic gradient at the aortic isthmus (mean, SD mmHg)	46.6 ± 19.3	57.0 ± 18.7	0.09
Peak velocity at the isthmus (mean, SD m/s)			
Inotropic and vasopressor support (n, %)	2 (12%)	6 (25%)	0.31
Inotropic support (n, %)	3 (18%)	7 (29%)	0.38
**Biomarkers (plasma concentration)**			
Renin (mean, SD pg/mL)	22 ± 7	35 ± 10	0.01
vWF (mean, SD UI/mL)	140 ± 30	170 ± 40	0.03
IL-6 (mean, SD, pg/mL)	4.5 ± 1.8	6.9 ± 2.3	0.02
TNF-alpha (mean, SD, pg/mL)	4.8 ± 1.7	7.2 ± 2.1	0.01
IL-6/TNF-alpha over 2	2 (7.7%)	14 (87.5%)	0.03
**Intraoperative**			
Duration of surgery (mean, SD min)	130 ± 20	140 ± 25	0.17
Aortic clamp time (mean, SD min)	25 ± 6	27 ± 5	0.36
Surgery under one month (n, %)	6 (35%)	7 (29%)	0.68
Extracorporeal circulation (n, %)	0 (0%)	1 (6.25%)	0.82
**Postoperative**			
Peak systolic gradient at the aortic isthmus (mean, SD m/s)	12 ± 6.27	13 ± 3.28	0.39
Peak velocity (mean, SD m/s)	1.7 ± 0.4	1.9 ± 0.2	0.46
Inotropic and/or vasopressor support (n, %)	2 (12%)	5 (21%)	0.41
Chylothorax (n, %)	0 (0%)	2 (8%)	0.18
Superficial wound infection (n,%)	1 (6%)	3 (12%)	0.52
Cardiogenic shock (n, %)	0 (0%)	2 (8%)	0.18

**Table 3:** Results of logistic regression.

	Univariate Analysis			Multivariable Analysis		
	OR	95% CI	*p*	OR	95% CI	*p*
IL-6/TNF-alpha ratio	7.4	2.4–22.9	<0.001	5.2	1.6–17.1	0.02
Renin plasma concentration	3.30	1.1–10.5	0.05	2.4	1.1–5.9	0.03
Gothic arch	4.80	1.2–20.9	0.03			
Aortic clamp time	3.60	2.1–13.2	0.045	3	1.2–9.2	0.046
Age	1.04	1.5–26.4	0.040			

Odds ratios for age are expressed per 1-year increase; odds ratios for plasma renin concentration are expressed per 1 pg/mL increase.

## Data Availability

Data are available upon request.
